# Development of a Real-Time Enzymatic Recombinase Amplification Assay (RT-ERA) and an ERA Combined with a Lateral Flow Dipstick (LFD) Assay (ERA-LFD) for Enteric Microsporidian (*Enterospora epinepheli*) in Grouper Fishes

**DOI:** 10.3390/biology14040330

**Published:** 2025-03-25

**Authors:** Minqi Chen, Yongcan Zhou, Shifeng Wang, Jian Luo, Weiliang Guo, Hengwei Deng, Pei Zheng, Zhihong Zhong, Baofeng Su, Dongdong Zhang, Zhi Ye

**Affiliations:** 1 School of Breeding and Multiplication (Sanya Institute of Breeding and Multiplication), Hainan University, Sanya 572025, China; 2 Hainan Provincial Key Laboratory for Tropical Hydrobiology and Biotechnology, School of Marine Biology and Fisheries, Collaborative Innovation Center of Marine Science and Technology, Hainan University, Haikou 570228, China; 3 Hainan Haiwangxing Aquatic Science and Technology Co., Ltd., Wenchang 571322, China; 4 School of Fisheries, Aquaculture and Aquatic Sciences, Auburn University, Auburn, AL 36849, USA; 5 MOE Key Laboratory of Marine Genetics and Breeding, College of Marine Life Sciences, Ocean University of China, Qingdao 266071, China; 6 Key Laboratory of Tropical Aquatic Germplasm of Hainan Province, Sanya Oceanographic Institution, Ocean University of China, Sanya 572025, China

**Keywords:** grouper (*Epinephelus* spp.), *Enterospora epinepheli*, enzymatic recombinase amplification (ERA), real-time ERA, ERA with a lateral flow dipstick

## Abstract

*Enterospora epinepheli* poses a severe threat to grouper aquaculture due to the absence of effective prevention and treatment strategies. To address this challenge, we developed and validated two isothermal diagnostic tools, the real-time enzymatic recombinase amplification (RT-ERA) assay and the enzymatic recombinase amplification combined with a lateral flow dipstick (ERA-LFD) assay, targeting the 18S rDNA gene of the parasite. These assays operate under isothermal conditions at ≤40 °C and offer rapid detection, with RT-ERA yielding results in 14~20 min and ERA-LFD in approximately 10 min. The RT-ERA assay demonstrated a strong linear relationship between plasmid copy numbers and cycle threshold (Ct) values (y = −2.1226x + 19.562, R^2^ = 0.9915), enabling accurate quantification. Both methods displayed a detection limit of 2 × 10^0^ copies/μL and no cross-reactivity with other aquaculture pathogens. Validation using grouper tissue and water samples from Hainan, China, demonstrated 100% concordance rates with basic ERA and outperformed compared to conventional PCR. These assays provide sensitive, specific, and rapid detection tools for effective monitoring and pathogen load assessment of *E. epinepheli*, with broad applicability to pathogen detection in aquaculture systems.

## Simple Summary

*Enterospora epinepheli* is a microsporidian parasite that poses a severe threat to the grouper farming industry, with limited options for prevention and treatment. Regular monitoring and early diagnosis are essential for managing infections. In this study, two rapid diagnostic methods were developed: a real-time enzymatic recombinase amplification (RT-ERA) assay and an enzymatic recombinase amplification combined with a lateral flow dipstick (ERA-LFD) assay, both targeting the 18S rDNA gene of *E. epinepheli*. These methods operate under isothermal conditions (≤40 °C) and offer rapid detection, with RT-ERA achieving results within 14~20 min and ERA-LFD within approximately 10 min. The detection limit for both methods was 2 × 10^0^ copies/μL, with high specificity and no cross-reactivity with other aquaculture pathogens. Validation with grouper tissue and aquaculture water samples from Hainan, China, showed 100% concordance with basic ERA and superior performance compared to conventional PCR. These assays provide effective tools for early diagnosis, pathogen load quantification, and environmental monitoring, contributing to the prevention and control of *E. epinepheli* infections in aquaculture.

## 1. Introduction

Groupers (*Epinephelus* spp.) are economically significant marine fish species predominantly distributed in the South China Sea [1]. However, the grouper aquaculture industry has been severely impacted in recent years by enteritic microsporidian infections, particularly in hatchery-bred juveniles [2,3,4]. The pathogen responsible was initially identified in intestinal samples from juvenile groupers exhibiting severe emaciation at a hatchery in South China [2]. Subsequently, it was characterized and designated as *Enterospora epinepheli* [3,4]. This pathogen primarily targets the intestinal epithelium of its host, causing clinical symptoms such as anorexia, emaciation, white feces syndrome, and, ultimately, high mortality. Pathological effects may also extend to the liver and other organs.

In addition, another three microsporidian species have been reported to infect grouper: *Glugea epinephelusis* [5], *Pleistophora* sp. [6], and *Glugea arabica* [7]. Among these, *G. arabica* also infects the intestinal wall of groupers. However, only *E. epinepheli* proliferates within the host cell nucleus, suggesting that it is more challenging to treat and control.

The prevalence of enteritic microsporidian infection spans key grouper farming regions, with horizontal transmission occurring among fish populations. The associated mortality rate can reach up to 100%, making it a most devastating disease in grouper aquaculture and resulting in significant economic losses in the industry [2,3,4]. Due to its intracellular lifestyle within the intestinal epithelium, the pathogen is highly concealed, and there are currently no effective prevention or treatment strategies. This underscores the importance of regular monitoring and early, accurate diagnosis for disease management.

Existing detection techniques for *E. epinepheli* include optical microscopy (OM) [2], electron microscopy (EM) [3], polymerase chain reaction (PCR) [2,3], and quantitative PCR (qPCR) using TaqMan probes [4]. Although OM and EM are valuable for morphological studies, their practical application is hindered by lengthy observation times, slow response rates, complex procedures, and limited sensitivity and specificity. While TaqMan qPCR offers high sensitivity and accuracy, its reliance on specialized laboratory equipment and controlled environments restricts its utility for rapid testing and early diagnosis in resource-limited settings [4,8].

To address these challenges, this study aims to develop an efficient, sensitive, and user-friendly detection method for the early diagnosis and rapid screening of *E. epinepheli* in groupers. Enzymatic recombinase amplification (ERA), a novel isothermal nucleic acid amplification technique, has shown promise in the detection of aquatic pathogens due to its simplicity, speed, and efficiency [9,10,11,12]. However, its application for detecting *E. epinepheli* in groupers has not yet been reported. ERA circumvents the need for large instruments required by traditional PCR, enabling rapid nucleic acid amplification under isothermal conditions (25–42 °C), with amplification efficiencies reaching billions of copies within several min. Real-time enzymatic recombinase amplification (RT-ERA) facilitates the rapid quantification of pathogens, allowing for disease risk assessment in a short timeframe. Additionally, ERA combined with a lateral flow dipstick (ERA-LFD) assay enables visual detection of results within 10 min, making it particularly suitable for on-site testing without requiring precision temperature-control equipment. LFD may also encounter troubleshooting issues like other lateral flow assays, including false results, background noise, and irregular or slow flow [13]. LFD false positives often result from primer dimer formation or the delayed release of gold conjugates [14]. To mitigate these issues, various studies have introduced novel conjugate blocking agents, such as MorffiTM, bovine serum albumin (BSA), and milk powder, which help reduce non-specific binding and enhance the overall sensitivity of lateral flow assays [15,16]. The partitioned isolation operation can also help reduce the occurrence of false positives to some extent.

In this study, the 18S rDNA gene of *E. epinepheli* was targeted to establish two rapid and accurate detection methods: RT-ERA and ERA-LFD. These assays were further applied to tissue and water samples collected from field settings to evaluate their clinical applicability. The findings of this research offer valuable tools for the early detection and prevention of enteritic microsporidian infections in groupers and provide a reference framework for detecting other aquatic diseases.

## 2. Materials and Methods

### 2.1. Fish Samples and Pathogens

Pathogens of *E. epinepheli*, *Amyloodinium ocellatum*, *Cryptocaryon irritans*, *Vibrio alginolyticus*, *V. harveyi*, *V. Parahaemolyticus*, *Photobacterium damselae* subsp. *damselae* (PDD), *Streptococcus iniae*, viral nervous necrosis (VNN), and Singapore grouper iridovirus (SGIV) have all been kept in our laboratory.

**Figure 1:**
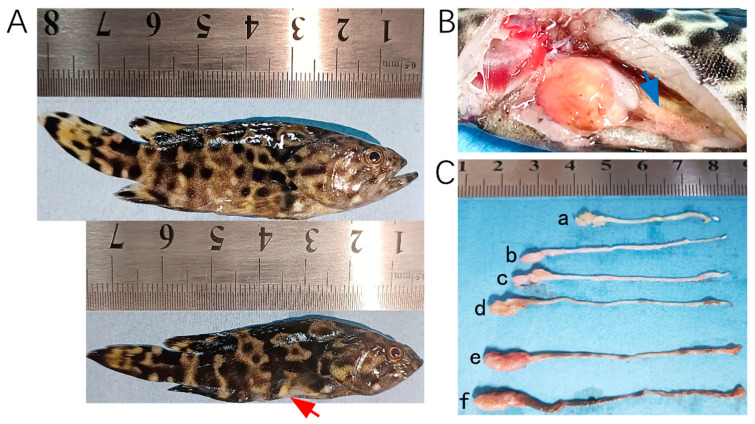
Hybrid groupers (*E. fuscoguttatus*♀ × *E. lanceolatus*♂) infected by *E. epinepheli*. (**A**): Relatively healthy grouper (top) and severe diseased grouper (bottom). (**B**): Intestinal content discharge from a diseased grouper. (**C**): Intestinal conditions of diseased grouper (a, b, c, and d), showing thinning and transparency of intestinal walls, residual mass content, and white feces discharge, and relatively healthy groupers (e and f). Red arrow indicates abdominal concavity, and blue arrow indicates flattened intestine.

In 2023, tissue samples were collected from 28 hybrid groupers (*Epinephelus fuscoguttatus*♀ × *E. lanceolatus*♂) at a farm in Wenchang, China, following an outbreak of *E. epinepheli*. In 2024, additional samples were obtained, including 14 tissue samples from hybrid groupers and leopard coral groupers (*Plectropomus leopardus*) as well as 26 pond water samples from cement pond aquaculture systems in Chengmai, Danzhou, and Lingao. Photographs of fish exhibiting typical symptoms are presented in Figure 1.

### 2.2. ERA Primer and Probe Design

Four partial sequences of the 18S rDNA gene of *E. epinepheli* were retrieved from GenBank. Among these, sequences MH345732 and KR263870 are associated with relevant publications [2,4]. The sequences identified were evaluated by Clustal Omega 1.2.4 [17] and the NCBI blastn tool. The primers and probes were designed based on the conserved regions located within the targeting region of the published PCR primers. To maintain amplification efficiency, the target sequences for this study ranged in length from 100 to 300 bp. All sequence-related information has been archived in Supplementary File S1.

The primers were designed using Primer Premier 6.0 (Premier Biosoft International, Palo Alto, CA, USA) [18], following the guidelines provided in the ERA nucleic acid amplification kit (Suzhou GenDx Gene Technology Co., Ltd., Suzhou, China) for conducting ERA reactions. To ensure primer compatibility and avoid mismatches both within the primers themselves and between the primers and probes, the ERA primers were designed to be 29 to 35 base pairs long. To balance amplification efficiency, rapid detection, and prevention of primer dimer formation, the amplicon length was maintained between 100 to 250 bp. Optimal primer combinations were identified through comparative trials and selected for subsequent validation.

The fluorescent probe was designed with a FAM fluorophore at the 5’ end, positioned at the 30th base, and a BHQ1 quencher group at the 15th base on the 3’ end. A tetrahydrofuran (THF) spacer was incorporated to separate the fluorophore from the quencher, and a C3-Spacer block group was added to the 3’ end to prevent extension. For the dipstick probe, a similar design was employed: a FAM fluorophore at the 5’ end, THF at the 30th base, and a C3-Spacer block group at the 3’ end. Both probes were 46 base pairs long to optimize detection performance.

A total of 24 primer combinations were tested, with purification carried out using polyacrylamide gel electrophoresis (PAGE) or high-performance liquid chromatography (HPLC), denoted as “PAGE” and “HPLC”, respectively. The details of the primers and probes are provided in Table S1.

### 2.3. The Construction of the Positive Standard Recombinant Plasmid

Using *E. epinepheli*-positive intestinal tissue samples as templates, we amplified conserved sequences within the 18S rDNA gene of *E. epinepheli* using the primer combination EEP-HPLC-F2/EEP-HPLC-R2, as detailed in Table S1. The amplified fragments were purified using the FastPure Gel DNA Extraction Mini Kit (DC301–01) from Nanjing Vazyme Biotechnology Co., Ltd., Nanjing, China. The purified products were ligated into the PEASY-T1Sample vector (CT111) purchased from Beijing Allgen Biotechnology Co., Ltd., Beijing, China, to construct the recombinant plasmid EEP-XL-2018a.

The recombinant plasmid was transformed into Escherichia coli DH5α competent cells (ZC101), obtained from Beijing Zhuangmeng International Bio-Gene Technology Co., Ltd., Beijing, China, and plated on LB agar containing ampicillin. The plates were incubated at 37 °C until colonies formed. Positive colonies were identified through dilution in enzyme-free sterile water and screened using M13 vector primers, followed by colony PCR. The identity of positive clones was confirmed by sequencing.

Positive clone strains were cultivated in LB liquid medium overnight, and plasmid DNA was extracted using the Omega Plasmid Mini Kit I DNA extraction kit (D6943) from Omega Bio-Tek, Norcross, GA, USA. The concentration of the extracted plasmid DNA was measured as 123 ng/μL using a Shanghai Yidian spectrophotometer (Shanghai Instrument and Electrical Analytical Instruments Co., Ltd., Shanghai, China). The plasmid copy number was calculated to be 2 × 10^10^ copies/μL based on Avogadro’s number [19]. The plasmid DNA was stored at −20 °C for subsequent use in ERA detection experiments.

### 2.4. DNA Extraction

Total DNA extraction from tissues was conducted following the manufacturer’s instructions of the OMEGA E.Z.N.A. Tissue DNA Kit D3396 (Omega Bio-Tek, Norcross, GA, USA). Approximately 30 mg of each sample was taken for the extraction of total tissue DNA, followed by the determination of DNA concentration using a Nanodrop microspectrophotometer, and standardized to a concentration of 200 ng/μL (re-extracted if the concentration was too low or diluted with the Elution Buffer from the kit if the concentration was too high). The eluted DNA was stored at −20 °C.

For total DNA extraction from water samples, 5 L of water was first filtered through a 0.22 μm micropore filter. The resulting filter membrane was cut into four pieces and placed in a clean 50 mL centrifuge tube. Total DNA extraction was then performed according to the OMEGA E.Z.N.A. Water DNA Kit (D5525-01; Omega Bio-Tek, Norcross, GA, USA) protocol. DNA concentrations were measured using a Nanodrop spectrophotometer and adjusted to 50 ng/μL. Samples with insufficient DNA concentrations were re-extracted, while those with excessive concentrations were diluted using the kit’s Elution Buffer. The purified DNA was stored at −20 °C.

### 2.5. Optimization of Primer Combination for Basic ERA Detection of E. epinepheli

Basic enzymatic recombinase amplification (ERA) was performed using the GenDx ERA KS101 nucleic acid amplification kit (Suzhou GenDx Gene Technology Co., Ltd., Suzhou, China). The reaction mixture for ERA consisted of 2.5 μL of forward primer (10 μM), 2.5 μL of reverse primer (10 μM), 2 μL of template DNA, 20 μL of solvent, and 21 μL of enzyme-free water, resulting in a total volume of 48 μL. This mixture was added to each amplification reagent tube, followed by the addition of 2 μL of ERA activator containing Mg^2+^. The tubes were then rapidly centrifuged and thoroughly mixed.

To optimize the primer combination, a basic ERA test was conducted using a plasmid template with a concentration of 2 × 10^5^ copies/μL. The reaction was performed with 24 different primer pairs (listed in Table S2) under the kit-recommended conditions of 39 °C for 15 min. Primers yielding high-intensity bands without primer dimers were selected for subsequent experiments.

### 2.6. Development of the RT-ERA Detection Method for E. epinepheli

The RT-ERA method utilizes the GenDx ERA Fluorescent Nucleic Acid Amplification Kit KS103 (Suzhou GenDx Gene Technology Co., Ltd., Suzhou, China). The reaction system includes a 48 μL premix containing 2.1 μL of forward primer (10 μM), 2.1 μL of reverse primer (10 μM), 0.6 μL of probe (10 μM), 2 μL of template DNA, 20 μL of solvent, and 21.2 μL of enzyme-free water. A total of 48 μL of the premix is initially added to each amplification reagent tube, followed by 2 μL of ERA activator containing Mg^2+^, which is added to the tube cap. The tubes are then rapidly centrifuged and thoroughly mixed. Fluorescence values are measured using the Q2000B Real-Time Fluorescence Quantitative PCR Instrument (Hangzhou Longji Technology Instrument Co., Ltd., Hangzhou, China). The experimental settings include absolute quantification as the type, TaqMan Reagents as the dye, and a reaction program of 40 cycles, with fluorescence values recorded every 30 s.

The amplification curve for the positive template exhibits an “S” shape, while no amplification is observed for the negative control. The Ct value is determined at the intersection of the amplification curve, with a threshold set at 300 CFU. The slope of the curve during the logarithmic phase indicates its steepness, while the fluorescence intensity at the plateau phase reflects the final fluorescence value.

Primer and Probe Selection: The optimal primer pair, EEP-HPLC-F2/EEP-HPLC-R2, identified in the basic ERA method (Section 2.2) is used for RT-ERA. The best probe, EEP-probe2a, is designed following the ERA probe design principles (Section 2.2).

Optimal Reaction Temperature: To determine the optimal temperature, reactions are performed using a plasmid template at a concentration of 2 × 10^5^ copies/μL at five temperatures: 33 °C, 35 °C, 37 °C, 39 °C, and 41 °C. The temperature yielding the lowest Ct value, steepest slope, and highest final fluorescence intensity is selected. The optimal reaction time is determined by identifying the cycle range at which the amplification curve plateaus.

### 2.7. Development of the ERA-LFD Detection Method for E. epinepheli

The ERA-LFD utilizes the GenDx ERA test paper nucleic acid amplification kit KS105 and the lateral flow test strip TS101 (Suzhou GenDx Biotech Co., Ltd., Suzhou, China). The primers and probes used in the ERA-LFD are listed in Table S1. The reaction system consists of a mixture of forward primer 2.1 μL (10 μM), reverse primer 2.1 μL (10 μM), probe 0.6 μL (10 μM), template DNA 2 μL, diluent 20 μL, and enzyme-free water 21.2 μL, totaling 48 μL of the premix. Initially, 48 μL of the premix is added to each amplification reagent tube. Subsequently, 2 μL of ERA activator (containing Mg^2+^) is added to the tube, followed by rapid centrifugation and mixing. After that, 2 μL Proteinase K (5 μg/100 μL) is used to terminate the reaction, according to the manufacturer’s instructions.

To observe the reaction effect using the test strip, the amplification product is diluted with enzyme-free water at a ratio of 40:1 (i.e., 5 μL of the reaction product is transferred into a 1.5 mL centrifuge tube and diluted with 200 μL of pure water). The test strip is then inserted into the centrifuge tube, and the results are read after 7~10 min. A strip displaying only one visible blue line in the control area will be considered negative, whereas a strip showing two visible lines, one in the control area and one in the test area (red color), will be considered positive. The reaction intensity is determined by the color intensity of the test line, with a darker red color indicating stronger reaction intensity.

Primer and Probe Selection: The optimal primer pair, EEP-HPLC-F2/EEP-HPLC-R2 (from the basic ERA method, Section 2.2), is used for the ERA-LFD method. The best reverse primer, EEP-R2-biotin, and probe, EEP-Sprobe2a, are designed according to the ERA primer and probe design principles (Section 2.2).

Determination of Optimal Reaction Temperature: To determine the optimal reaction temperature, reactions are performed at four different temperatures: 37 °C, 38 °C, 39 °C, and 40 °C. The temperature that yields the most distinct red color of the test line is selected as the optimal temperature.

In order to avoid a false positive or template contamination, partitioned isolation operation was employed. The reagent area, the amplification area, and the detection area need to be strictly separated. Before leaving the first two areas, ensure that the test tubes are tightly sealed. During the final detection, the test strips, which have absorbed the reaction solution, should not be placed together until they are completely dry.

### 2.8. Sensitivity Analysis

Gradient dilutions of recombinant plasmid standards were prepared, with concentration gradients ranging from 2 × 10^0^ to 2 × 10^8^ copies/μL used as templates for the reactions. Both RT-ERA and ERA-LFD, which had been optimized for temperature conditions, were utilized for the reactions and compared with the results from conventional PCR and basic ERA. For conventional PCR, the primers EEP-PAGE-F2/EEP-PAGE-R2 (Table S1) were used in a reaction system (50 μL) comprising 2 μL (10 μL) of forward primer, 2 μL (10 μL) of reverse primer, 2 μL of template DNA, 25 μL of 2 × Rapid Taq Master Mix (Nanjing Vazyme Biotechnology Co., Ltd., Nanjing, China), and ddH_2_O to make up to 50 μL. DNA denaturation was carried out at 95 °C for 5 min, followed by a total of 35 PCR cycles under the following conditions: DNA denaturation at 94 °C for 30 s, primer annealing at 57 °C for 30 s, and DNA polymerization at 72 °C for 1 min. After the final cycle, the reactions were terminated at 72 °C for 10 min. For the basic ERA, the primers EEP-HPLC-F2/EEP-HPLC-R2 (Table S1) were used with a reaction temperature and time of 39 °C for 15 min. The RT-ERA employed a combination of EEP-HPLC-F2/EEP-HPLC-R2/EEP-probe2a (Table S1) with a reaction temperature and time of 39 °C for 14 min. The ERA-LFD used a combination of EEP-HPLC-F2/EEP-R2-biotin/EEP-Sprobe2a (Table S1) with a reaction temperature and time of 40 °C for 10 min.

### 2.9. Specificity Analysis

Using DNA from *E. epinepheli*-positive tissue as well as DNA from *A. ocellatum*, *C. irritans*, *V. alginolyticus*, *V. harvey*, *V. parahaemolyticus*, *Photobacterium damselae* subsp. damselae (PDD), *S. iniae*, viral nervous necrosis (VNN), and Singapore grouper iridovirus (SGIV) as templates, reactions were conducted employing the optimized temperature conditions for RT-ERA and the optimized temperature and time conditions for ERA-LFD. DNA concentrations of these pathogens were also standardized to a concentration of 200 ng/μL as indicated in the DNA extraction procedures. The RT-ERA employed a combination of EEP-HPLC-F2/EEP-HPLC-R2/EEP-probe2a (Table S1) with a reaction temperature and time of 39 °C for 14 min. The ERA-LFD used a combination of EEP-HPLC-F2/EEP-R2-biotin/EEP-Sprobe2a (Table S1) with a reaction temperature and time of 40 °C for 10 min.

### 2.10. Clinical Tissue Samples and Water Samples for E. epinepheli Detection

To evaluate the practical application of *E. epinepheli* detection in clinical samples, we employed RT-ERA, which had been optimized for temperature conditions, and ERA-LFD, optimized for both temperature and time conditions, alongside the basic ERA and conventional PCR detection methods. These methods were applied to detect *E. epinepheli* in a total of 42 tissue samples and 26 water samples collected between 2023 and 2024, as described previously, to assess the potential of RT-ERA and ERA-LFD for clinical applications. The conventional PCR and the basic ERA procedures have been explained in Section 2.8.

## 3. Results

### 3.1. Optimization of the Primer Selection by ERA

**Figure 2:**
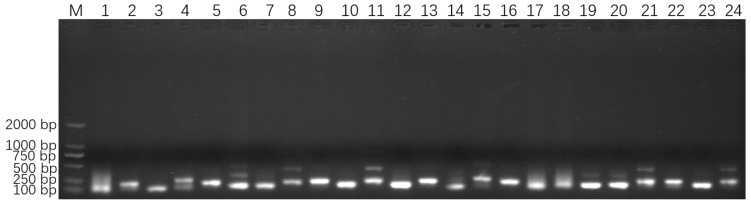
Optimized primer screening results for *E. epinepheli.* M: Maker D2000, 1~24: primer pairs from group 1 to 24 in Table S1.

The four 18S rDNA genes of *E. epinepheli* have greater than 99.6% identity, and the primers and probes were located within their conserved regions (Supplementary File S1). In the ERA primer screening experiment, a recombinant plasmid standard at a concentration of 2 × 10^5^ copies/μL was used as a positive template. All primer combinations listed in Table S1 successfully amplified the target sequence. As shown in Figure 2, primer combinations 1, 2, 4, 6–9, 11, 14, 15, 17–22, and 24 produced non-specific bands; primer combinations 3, 5, and 16 yielded faint bands; primer combinations 10, 12, and 23 generated smaller-sized bands. These issues led to the exclusion of these primer combinations from further analysis. Ultimately, primer combination 13 (EEP-HPLC-F2/EEP-HPLC-R2), which amplifies a specific target product of 206 bp, was selected. Furthermore, HPLC purification of the primers resulted in brighter bands compared to PAGE purification, confirming the effectiveness of the HPLC-purified primers. Consequently, the optimal primer combination identified during the ERA primer screening experiment, EEP-HPLC-F2/EEP-HPLC-R2, was utilized in the following RT-ERA and ERA-LFD experiments.

### 3.2. Optimization of ERA Assay Conditions for the Detection

**Figure 3:**
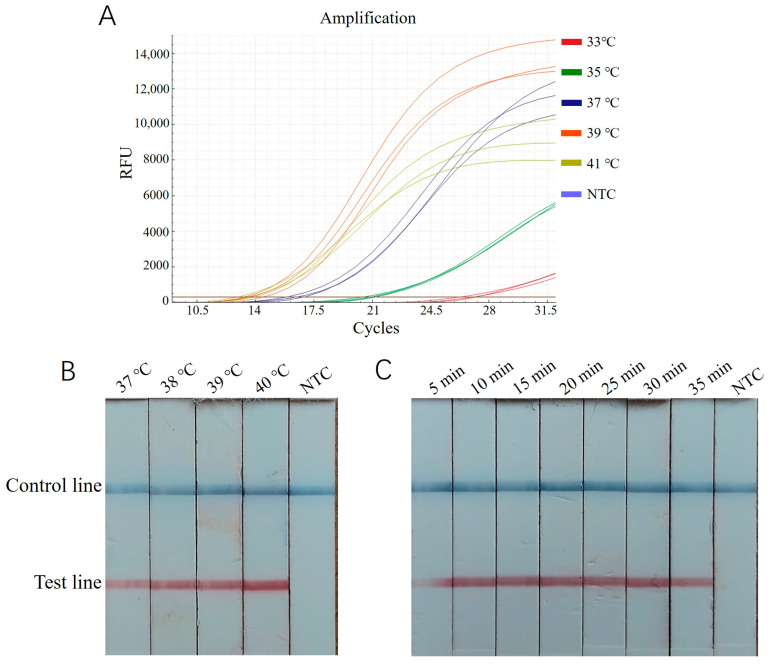
Optimization of ERA reaction condition for *E. epinepheli* detection. (**A**): RT-ERA temperature optimization test. (**B**): ERA-LFD temperature optimization test. (**C**): ERA-LFD time optimization test. Three replicates were performed for each RT-ERA temperature. In ERA-LFD, a blue control line indicates a negative result, while both a blue control and red test line indicate a positive result. Reaction intensity is determined by the test line’s red color intensity. NTC, Negative control.

The RT-ERA method employed the primers EEP-HPLC-F2/EEP-HPLC-R2 (25) selected in 3.1 and the probe EEP-probe2a (26). Using a recombinant plasmid standard at a concentration of 2 × 10^5^ copies/μL as a positive template, reactions were conducted at 33 °C, 35 °C, 37 °C, 39 °C, and 41 °C for 20 min. The results showed that the amplification curve at 39 °C exhibited the lowest Ct value, the steepest slope, and the highest fluorescence intensity (Figure 3A). Thus, 39 °C was chosen as the optimal reaction temperature. Additionally, the interval where the cycle number reaches a plateau (between approximately 28 and 40 cycles) corresponded to an optimal reaction time range of 14~20 min.

For the ERA-LFD assay, the same recombinant plasmid standard (2 × 10^5^ copies/μL) was used as a positive template. Reactions were performed at 37 °C, 38 °C, 39 °C, and 40 °C for 20 min, followed by diluting 5 μL of the reaction mixture in 200 μL of enzyme-free water and testing with a lateral flow strip. As shown in Figure 3B, the intensity of the control and test bands was most pronounced at 40 °C, making this the optimal reaction temperature. Subsequently, reactions were tested for durations of 5, 10, 15, 20, 25, 30, and 35 min. The bands were faint at 5 min, while, from 10 to 35 min, they were clearly visible, with no significant increase in intensity over time (Figure 3C). Therefore, 10 min was identified as the optimum time for color development.

### 3.3. Sensitivity Analysis Result

**Figure 4:**
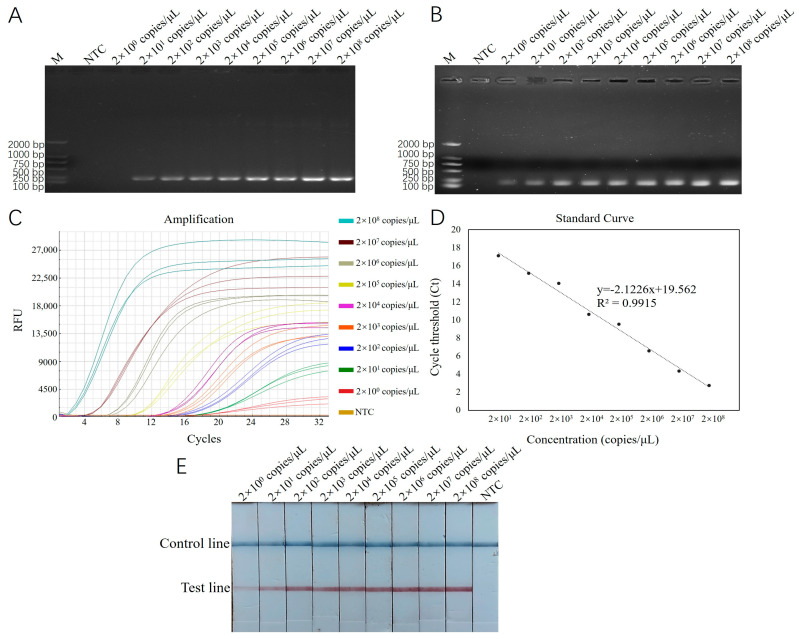
(**A**): Sensitivity testing results for *E. epinepheli* PCR sensitivity test. (**B**): Basic ERA sensitivity test. (**C**): RT-ERA sensitivity test. (**D**): RT-ERA standard curve. (**E**): ERA-LFD sensitivity test. M: Marker D2000. NTC: negative control.

Using recombinant plasmid standards with concentrations ranging from 2 × 10^1^ to 2 × 10^8^ copies/μL as templates, reactions were carried out with RT-ERA and ERA-LFD, which had been optimized for both reaction conditions. The results were then compared with those from conventional PCR and basic ERA methods. The results of the conventional PCR detection displayed bands across the concentration range from 2 × 10^1^ to 2 × 10^8^ copies/μL, with a detection limit of 2 × 10^1^ copies/μL (Figure 4A). The results of the basic ERA detection produced bands across the concentration range from 2 × 10^0^ to 2 × 10^8^ copies/μL, with a detection limit of 2 × 10^0^ copies/μL (Figure 4B). For RT-ERA detection, the amplification curves for three biological replicates were observed at each concentration gradient, establishing the detection limit of the RT-ERA method developed in this study as 2 × 10^0^ copies/mL (Figure 4C). A standard curve was generated based on the measured relationship between the copy number of recombinant plasmids (x) and the threshold cycle number (Ct value; y). The fitted equation for the curve is y = −2.1226 x + 19.562 (Figure 4D), with a correlation coefficient (R^2^) of 0.9915. The ERA-LFD detection results exhibited red test lines on the lateral flow strips within the concentration range from 2 × 10^0^ to 2 × 10^8^ copies/μL, confirming the detection limit of the ERA-LFD method as 2 × 10^0^ copies/μL (Figure 4E).

### 3.4. Specificity Analysis Result

**Figure 5:**
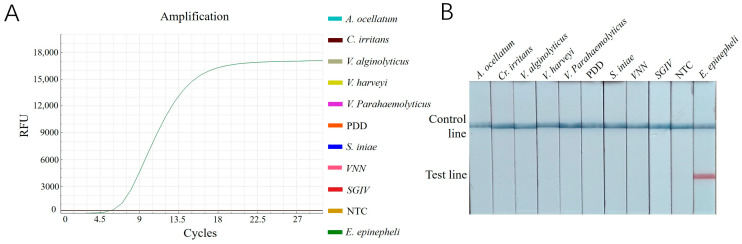
(**A**): Specificity testing results for *E. epinepheli* RT-ERA specific experiment. (**B**): ERA-LFD specific experiment. PDD, *Photobacterium damselae* subsp. *damselae*; VNN: viral nervous necrosis; SGIV: Singapore grouper iridovirus; NTC: negative control.

The results revealed that only the reactions with DNA samples of *E. epinepheli* as templates produced amplification curves in the RT-ERA assay (Figure 5A) and a positive red band on the lateral flow dipsticks (Figure 5B). All other pathogens and negative controls showed negative results. These findings demonstrate that both established methods exhibit high specificity for detecting *E. epinepheli*.

### 3.5. Detection of Clinical Tissue Samples and Water Samples

A total of 42 tissue samples (Table S2) and 26 water samples (Table S3) collected from 2023 to 2024 were subjected to RT-ERA and ERA-LFD for the detection of *E. epinepheli* in groupers. The results were compared with those from conventional PCR and basic ERA methods. The positivity rates for the four methods—conventional PCR, basic ERA, RT-ERA, and ERA-LFD—are statistically summarized in Table 1. The agarose gel electrophoresis images, fluorescent amplification curves, and test strip results for each detection method are displayed in Figure 5, respectively.

Conventional PCR detected 22 positive samples: a total of 21 samples from the F1 farm (emaciated tissue samples from Wenchang) and one tissue sample from the F10P3 farm in Danzhou (Figure S1). The basic ERA method identified 29 positive samples (Figure S2). The RT-ERA method detected 29 positive samples and qualified the *E. epinepheli* numbers based on Ct values (Figure S3). The ERA-LFD method identified 29 positive samples (Figure S4). Among these, 23 positive tissue samples were from the F1 farm (emaciated tissue samples from Wenchang; Table S2), and three positive samples each of tissue and water were from the F8P1, F10P1, and F10P3 farms in Danzhou (Table S2, Table S3). The positive detection rates for tissue samples and water samples were as follows: RT-ERA, ERA-LFD, and basic ERA showed consistent results, with a positive rate of 61.9% for tissue samples and 11.5% for water samples; conventional PCR yielded positive rates of 52.4% for tissue samples and 0% for water samples (Table 1).

## 4. Discussion

The microsporidian parasite *E. epinepheli*, first reported in China in 2016, primarily affects juvenile fish, causing intestinal inflammation and dysbiosis [2,3,4]. As documented in the literature, its congeneric species have exerted a significant influence on a multitude of aquatic animals, such as *Cancer pagurus* [20], *Litopenaeus vannamei* [21], *Oncorhynchus tshawytscha* [22], and *Sparus aurata* [23,24]. This acute disease is challenging to detect in its early stages and can result in high mortality rates within 2 to 3 days after clinical symptoms appear. Its concealment within host intestinal cells exacerbates the difficulty of controlling and preventing it. Therefore, early, rapid, and accurate detection is essential for the prevention and control of *E. epinepheli* infections.

Previous research has established conventional PCR [2] and TaqMan probe-based qPCR [4] assays for *E. epinepheli* detection, both of which require expensive specialized equipment and longer detection times. In contrast, the RT-ERA and ERA-LFD assays developed in this study offer highly efficient alternatives to existing methods and have already been applied to several aquaculture species [9,10,11,12,25]. A total of 24 ERA primer combinations targeting highly conserved sequences of the *E. epinepheli* 18S rDNA gene were designed, comprising 11 PAGE-purified and 13 HPLC-purified primers. Among these, the primer pair EEP-HPLC-F2/EEP-HPLC-R2 exhibited the highest amplification efficiency in ERA and was selected for use in the RT-ERA and ERA-LFD assays. Both probes were identically positioned, demonstrating equivalent sensitivity and specificity. The RT-ERA reaction was effective across a temperature range of 33–41 °C, with an optimal temperature of 39 °C yielding the lowest Ct values, steepest slopes, and highest fluorescence intensities. Temperature optimization experiments indicated that the reaction plateaued within 28–40 cycles (14~20 min).

For ERA-LFD optimization, 40 °C was identified as the optimal assay temperature, balancing expediency and assay positivity. Reaction time optimization revealed that LFD test lines were detectable between 5 to 35 min, with the brightest positive band observed at 10 min at 40 °C, establishing this as the ideal reaction condition. The specificity of the RT-ERA and ERA-LFD assays was validated by testing against common grouper pathogens, with positive results observed exclusively for *E. epinepheli* in amplification curves and LFD bands. Furthermore, this study demonstrated that HPLC-purified primers were more sensitive than PAGE-purified primers in ERA, likely due to HPLC purification being unaffected by analyte volatility or thermal stability, thereby enhancing detection sensitivity. The conceptual framework for this experiment builds on the findings from previous studies [9,10,11,12,25,26].

ERA overcomes the effects of inhibitors during sample DNA preparation, achieving high sensitivity with a detection range of 1–2 copies/μL [9,10,11,12,25,26]. The RT-ERA, when combined with a portable fluorescence quantifier, provides accurate results within 14~20 min at 39 °C based on the quantification curve. Additionally, ERA-LFD offers results within 10 min at 40 °C, with easily interpretable test bands, providing greater convenience compared to the gel-based analysis used for ERA and PCR. In order to avoid a false positive or template contamination, partitioned isolation operation was employed. This approach contributes to the high accuracy of our ERA detection method.

Within a very short time and with lower reaction times, the established ERA assays demonstrated higher sensitivity compared to conventional PCR while achieving similar levels (less than 10 copies/μL) of performance to TaqMan-based qPCR [4]. Our ERA assay’s sensitivity reached a comparable level to other recent isothermal amplification techniques, such as recombinase polymerase amplification RPA [27,28,29] and Loop-Mediated Isothermal Amplification LAMP methods [30,31,32], in detecting aquaculture pathogens. An RPA-LFD assay for detecting Carp herpesvirus 3 was developed, which can detect as low as 10 copies of viral DNA within 20 min [33]. Similarly, An RPA-LFD method for detecting *Flavobacterium columnare* was established, with a detection limit of 0.4 CFU in artificially contaminated samples [34]. LAMP, known for its high sensitivity and rapid detection, can identify six copies of WSSV viral DNA in 55 min [32]. While the principle of RPA is similar to ERA and was developed somewhat earlier, their efficacy is comparable. However, although LAMP can achieve high sensitivity, its primer design is more complex, and it is prone to false positives [30].

It is worth noting that in sensitivity testing, high concentrations of the standard sample can generate very high amplification signals, making it unnecessary to include them in sensitivity assessments [35]. In addition, studies have reported the use of the CRISPR-Cas12a system in combination with isothermal amplification techniques for pathogen detection [36,37,38,39]. This approach shows advantages in both sensitivity and specificity, suggesting that future optimization of the detection system established in this study could be achieved by integrating CRISPR-Cas, allowing for faster, more convenient, and accurate detection in aquaculture.

This study is the first to monitor fish microsporidia through water samples using the ERA method, achieving promising detection results. It highlights the importance of an integrated detection strategy [40]. In this study, 68 samples were tested, including 28 tissue samples showing severe or mild emaciation symptoms, 14 tissue and water samples each with other clinical symptoms or from healthy fish, and 12 water samples without corresponding tissue samples. The positive detection rates for RT-ERA, ERA-LFD, and basic ERA were consistent (tissue samples: 61.9%, water samples: 11.5%), all outperforming conventional PCR (tissue samples: 52.4%, water samples: 0%). The superior sensitivity of ERA is attributed to its higher resistance to inhibitors during sample DNA preparation. Notably, emaciated tissue samples (F1) showed lower Ct values, higher fluorescence intensity, and brighter bands in RT-ERA and ERA-LFD compared to other clinical tissue samples (F8 and F10), indicating faster detection speeds. Quantitative analysis via RT-ERA revealed that tissue samples with pronounced symptoms of *E. epinepheli* infection had higher DNA copy numbers compared to samples with mild symptoms and water samples, enabling the analysis of disease progression. The lower DNA copy numbers in water samples may partly result from DNA extraction concentrations being one-quarter of those used for tissue samples.

Detection consistency between fish and water samples from the same farms (e.g., F8P1, F10P1, and F10P3) suggests that *E. epinepheli* can be horizontally transmitted through water. Continuous water monitoring and proactive preventive measures are crucial to minimizing aquaculture losses. This study introduces an effective method for predicting and monitoring *E. epinepheli* infections through pathogen concentration analysis in water samples. Early detection and intervention measures, such as treating juvenile fish or performing timely water changes to disrupt pathogen transmission, can prevent disease outbreaks. Importantly, this method enables non-invasive monitoring, thereby reducing aquaculture losses.

## 5. Conclusions

In conclusion, the RT-ERA and ERA-LFD detection methods for *E. epinepheli* established in this study are simple, rapid, highly sensitive, and specific, requiring no expensive or professional equipment. They lay a solid foundation for the rapid and accurate diagnosis of *E. epinepheli* infections both in fish and water samples, providing robust technical support for the sustainable development of the aquaculture industry.

## Figures and Tables

**Table 1:** Summary of *E. epinepheli* detection results by different assays in grouper tissues and environmental water samples from Hainan Province, China.

Detection Method	ERA/RT-ERA/ERA-LFD		PCR	
Sample	Tissue	Water	Tissue	Water
Positive number	26	3	22	0
Negative number	16	23	20	26
Total number	42	26	42	26
Positive rate	61.9%	11.5%	52.4%	0

## Data Availability

All data supporting the reported results are included within the manuscript and the supplementary materials.

## Supplementary Materials

The following supporting information can be downloaded at: https://www.mdpi.com/article/10.3390/biology14040330/s1, Table S1. Primer and probe information. Table S2. The *E. epinepheli* detection results of clinical fish samples from farms in different locations of Hainan Province, China. Table S3. The *E. epinepheli* detection results of environmental water samples from different locations of a farm in Hainan Province, China. Figure S1. *E. epinepheli* detection results conducted by PCR in 68 samples collected from grouper farms in Hainan, China. Figure S2. *E. epinepheli* detection results conducted by ERA in 68 samples collected from grouper farms in Hainan, China. Figure S3. *E. epinepheli* detection results conducted by RT-ERA. Detection results of *E. epinepheli* in 68 samples collected from grouper farms in Hainan, China. Figure S4. *E. epinepheli* detection results conducted by ERA-LFD in 68 samples collected from grouper farms in Hainan, China. Supplementary File S1. The *E. epinepheli* 18S rDNA sequence-related information.
