# Supplementary material for: Development of a Real-Time Enzymatic Recombinase Amplification Assay (RT-ERA) and an ERA Combined with a Lateral Flow Dipstick (LFD) Assay (ERA-LFD) for Enteric Microsporidian (Enterospora epinepheli) in Grouper Fishes
Source: Biology (Basel). 2025 Mar 25;14(4):330. doi: 10.3390/biology14040330 (PMC12025048; doi:10.3390/biology14040330)
Supplement: Supplementary file 1 [file biology-14-00330-s001.zip › biology-3461216-supplementary.pdf]

Table S1. Primers and probes information.

Experiment	No.	Primer/probe	Primer sequences（5′-3′）	Product length (bp)
Basic ERA	1	EEP-PAGE-F1	GTGGTATAGGTAGACAAAGAATGAAATC	139
		EEP-PAGE-R1	CTAGAATTACAGCGGTATCTAATCACTT	
	2	EEP-PAGE-F2	CTATGTGTAAAGTTATTGGAGGGTAAGT	206
		EEP-PAGE-R2	GAGATTTCATTCTTTGTCTACCTATACC	
	3	EEP-PAGE-Fa	GACTGAAACTTAAACGATATTGGCGGAAG	112
		EEP-PAGE-Ra	AATCAATCTACGATAAACTTGACCCTGGTAA	
	4	EEP-PAGE-Fb	GTGGATTATGCCGCTTAATTTAACTCAACG	245
		EEP-PAGE-Rb	TATTGTATTACGCTTGCTGCCCAGGATA	
	5	EEP-PAGE-Fc	CTATGTGTAAAGTTATTGGAGGGTAAGTTTGG	198
		EEP-PAGE-Rc	ATTCTTTGTCTACCTATACCACGTTCCC	
	6	Era-EEP-PAGE-F3	GGTCAAGGACTAAGGCTGGAGT	157
		Era-EEP-PAGE-R3	CAGTCTTGCGGCTGTACTATCC	
	7	Era-EEP-PAGE-F4	CGTGGTATAGGTAGACAAAGAATGAAATCTCT	143
		Era-EEP-PAGE-R4	CTGCTAGAATTACAGCGGTATCTAATCACTT	
	8	Era-EEP-PAGE-F5	GTAGTGGATTATGCCGCTTAATTTAACTCAAC	203
		Era-EEP-PAGE-R5	CCTGTTATCGTCTACACCTTCCTGTACTTAA	
	9	Era-EEP-PAGE-F6	CAGTAGTGGATTATGCCGCTTAATTTAACTCAAC	214
		Era-EEP-PAGE-R6	CATTACGGACCTGTTATCGTCTACACCTTC	
	10	Era-EEP-PAGE-F8	AAGTGATTAGATACCGCTGTAATTCTAGCAGTAA	154
		Era-EEP-PAGE-R8	CTTCCGCCAATATCGTTTAAGTTTCAGTCTT	
	11	Era-EEP-PAGE-F9	AGTGGATTATGCCGCTTAATTTAACTCAAC	201
		Era-EEP-PAGE-R9	CCTGTTATCGTCTACACCTTCCTGTACTTAA	
	12	EEP-HPLC-F1	GTGGTATAGGTAGACAAAGAATGAAATC	139
		EEP-HPLC-R1	CTAGAATTACAGCGGTATCTAATCACTT	
	13	EEP-HPLC-F2	CTATGTGTAAAGTTATTGGAGGGTAAGT	206
		EEP-HPLC-R2	GAGATTTCATTCTTTGTCTACCTATACC	
	14	EEP-HPLC-Fa	GACTGAAACTTAAACGATATTGGCGGAAG	112
		EEP-HPLC-Ra	AATCAATCTACGATAAACTTGACCCTGGTAA	
	15	EEP-HPLC-Fb	GTGGATTATGCCGCTTAATTTAACTCAACG	245
		EEP-HPLC-Rb	TATTGTATTACGCTTGCTGCCCAGGATA	
	16	EEP-HPLC-Fc	CTATGTGTAAAGTTATTGGAGGGTAAGTTTGG	198
		EEP-HPLC-Rc	ATTCTTTGTCTACCTATACCACGTTCCC	
	17	Era-EEP-HPLC-F1	GTAGTGGATTATGCCGCTTAATTTAACTCAAC	203
		Era-EEP-HPLC-R1	CCTGTTATCGTCTACACCTTCCTGTACTTAA	
	18	Era-EEP-HPLC-F2	AGTGGATTATGCCGCTTAATTTAACTCAAC	201
		Era-EEP-HPLC-R2	CCTGTTATCGTCTACACCTTCCTGTACTTAA	
	19	Era-EEP-HPLC-F3	GGTCAAGGACTAAGGCTGGAGT	157
		Era-EEP-HPLC-R3	CAGTCTTGCGGCTGTACTATCC	
	20	Era-EEP-HPLC-F4	CGTGGTATAGGTAGACAAAGAATGAAATCTCT	143
		Era-EEP-HPLC-R4	CTGCTAGAATTACAGCGGTATCTAATCACTT	
	21	Era-EEP-HPLC-F5	GTAGTGGATTATGCCGCTTAATTTAACTCAAC	203
		Era-EEP-HPLC-R5	CCTGTTATCGTCTACACCTTCCTGTACTTAA	

	22	Era-EEP-HPLC-F6	CAGTAGTGGATTATGCCGCTTAATTTAACT CAAC	214
		Era-EEP-HPLC-R6	CATTACGGACCTGTTATCGTCTACACCTTC	
	23	Era-EEP-HPLC-F8	AAGTGATTAGATACCGCTGTAATTCTAGCA GTAA	154
		Era-EEP-HPLC-R8	CTTCCGCCAATATCGTTTAAGTTTCAGTCTT	
	24	Era-EEP-HPLC-F9	AGTGGATTATGCCGCTTAATTTAACTCAAC	201
		Era-EEP-HPLC-R9	CCTGTTATCGTCTACACCTTCCTGTACTTAA	
RT-ERA	13	EEP-HPLC-F2	CTATGTGTAAAGTTATTGGAGGGTAAGT	206
		EEP-HPLC-R2	GAGATTTCATTCTTTGTCTACCTATACC	
	26	EEP-probe2a	TGGTTGTTGCAGTTAAAACGTCCGTAGCTG（FAM-dT）A（THF）A（BHQ1-dT）GCAATTAAAACACGA（C3-SPACER）	46
ERA-LFD	27	EEP-HPLC-F2	CTATGTGTAAAGTTATTGGAGGGTAAGT	206
		EEP-R2-biotin	Biotin-GAGATTTCATTCTTTGTCTACCTATACC	
	28	EEP-Sprobe2a	（FAM-dT）GTTGTTGCAGTTAAAACGTCCGTAGCTGTA（THF）ATGCAATTAAAACAC（C3-SPACER）	46
PCR	29	EEP-PAGE-F2	CTATGTGTAAAGTTATTGGAGGGTAAGT	206
		EEP-PAGE-R2	GAGATTTCATTCTTTGTCTACCTATACC	

Table S2. The *E. epinepheli* detection results of clinical fish samples from farms in different locations of Hainan Province, China.

Sample No.	PCR	ERA	RT-ERA	ERA-LFD	Pool No.	Farm No.	Location	Species
T1	+	+	+	+	P1	F1	Wenchang	Hybrid grouper
T2	+	+	+	+	P1	F1	Wenchang	Hybrid grouper
T3	+	+	+	+	P1	F1	Wenchang	Hybrid grouper
T4	+	+	+	+	P1	F1	Wenchang	Hybrid grouper
T5	+	+	+	+	P1	F1	Wenchang	Hybrid grouper
T6	+	+	+	+	P1	F1	Wenchang	Hybrid grouper
T7	+	+	+	+	P1	F1	Wenchang	Hybrid grouper
T8	-	-	-	-	P1	F1	Wenchang	Hybrid grouper
T9	+	+	+	+	P1	F1	Wenchang	Hybrid grouper
T10	+	+	+	+	P2	F1	Wenchang	Hybrid grouper
T11	+	+	+	+	P2	F1	Wenchang	Hybrid grouper
T12	-	-	-	-	P2	F1	Wenchang	Hybrid grouper
T13	-	-	-	-	P2	F1	Wenchang	Hybrid grouper
T14	-	-	-	-	P2	F1	Wenchang	Hybrid grouper
T15	-	-	-	-	P2	F1	Wenchang	Hybrid grouper
T16	+	+	+	+	P2	F1	Wenchang	Hybrid grouper
T17	+	+	+	+	P2	F1	Wenchang	Hybrid grouper
T18	+	+	+	+	P2	F1	Wenchang	Hybrid grouper
T19	+	+	+	+	P3	F1	Wenchang	Hybrid grouper
T20	+	+	+	+	P3	F1	Wenchang	Hybrid grouper
T21	-	+	+	+	P3	F1	Wenchang	Hybrid grouper
T22	+	+	+	+	P3	F1	Wenchang	Hybrid grouper
T23	+	+	+	+	P3	F1	Wenchang	Hybrid grouper
T24	+	+	+	+	P3	F1	Wenchang	Hybrid grouper
T25	+	+	+	+	P3	F1	Wenchang	Hybrid grouper
T26	+	+	+	+	P3	F1	Wenchang	Hybrid grouper
T27	-	+	+	+	P3	F1	Wenchang	Hybrid grouper
T28	+	+	+	+	P3	F1	Wenchang	Hybrid grouper
T29	-	-	-	-	P1	F2	Chengmai	*P. leopardus*
T30	-	-	-	-	P1	F5	Danzhou	Hybrid grouper
T31	-	-	-	-	P2	F5	Danzhou	Hybrid grouper

T32	-	-	-	-	P1	F7	Danzhou	Hybrid grouper
T33	-	-	-	-	P2	F7	Danzhou	Hybrid grouper
T34	-	-	-	-	P3	F7	Danzhou	Hybrid grouper
T35	-	+	+	+	P1	F8	Danzhou	*P. leopardus*
T36	-	-	-	-	P2	F8	Danzhou	*P. leopardus*
T37	-	-	-	-	P3	F8	Danzhou	*P. leopardus*
T38	-	+	+	+	P1	F10	Danzhou	Hybrid grouper
T39	-	-	-	-	P2	F10	Danzhou	Hybrid grouper
T40	+	+	+	+	P3	F10	Danzhou	Hybrid grouper
T41	-	-	-	-	P4	F10	Danzhou	*P. leopardus*
T42	-	-	-	-	P5	F10	Danzhou	*P. leopardus*

−, negative; +, positive.

Table S3. The *E. epinepheli* detection results of environmental water samples from different locations of farm in Hainan Province, China.

**Sample No.**	**PCR**	**ERA**	**RT-ERA**	**ERA-LFD**	**Pool No.**	**Farm No.**	**Location**	**Species**
W1	-	-	-	-	P1	F2	Chengmai	*P. leopardus*
W2	-	-	-	-	P2	F2	Chengmai	*P. leopardus*
W3	-	-	-	-	P1	F3	Lingao	Hybrid grouper
W4	-	-	-	-	P2	F3	Lingao	Hybrid grouper
W5	-	-	-	-	P1	F4	Danzhou	Hybrid grouper
W6	-	-	-	-	P2	F4	Danzhou	Hybrid grouper
W7	-	-	-	-	P3	F4	Danzhou	Hybrid grouper
W8	-	-	-	-	P4	F4	Danzhou	Hybrid grouper
W9	-	-	-	-	P1	F5	Danzhou	Hybrid grouper
W10	-	-	-	-	P2	F5	Danzhou	Hybrid grouper
W11	-	-	-	-	P3	F5	Danzhou	Hybrid grouper
W12	-	-	-	-	P4	F5	Danzhou	Hybrid grouper
W13	-	-	-	-	P1	F6	Danzhou	Hybrid grouper
W14	-	-	-	-	P2	F6	Danzhou	Hybrid grouper
W15	-	-	-	-	P1	F7	Danzhou	Hybrid grouper
W16	-	-	-	-	P2	F7	Danzhou	Hybrid grouper
W17	-	-	-	-	P3	F7	Danzhou	Hybrid grouper
W18	-	+	+	+	P1	F8	Danzhou	Hybrid grouper
W19	-	-	-	-	P2	F8	Danzhou	*P. leopardus*
W20	-	-	-	-	P3	F8	Danzhou	*P. leopardus*
W21	-	-	-	-	P1	F9	Danzhou	Hybrid grouper
W22	-	-	-	-	P2	F9	Danzhou	Hybrid grouper
W23	-	-	-	-	P3	F9	Danzhou	Hybrid grouper
W24	-	+	+	+	P1	F10	Danzhou	Hybrid grouper
W25	-	-	-	-	P2	F10	Danzhou	Hybrid grouper
W26	-	+	+	+	P3	F10	Danzhou	Hybrid grouper

−, negative; +, positive.

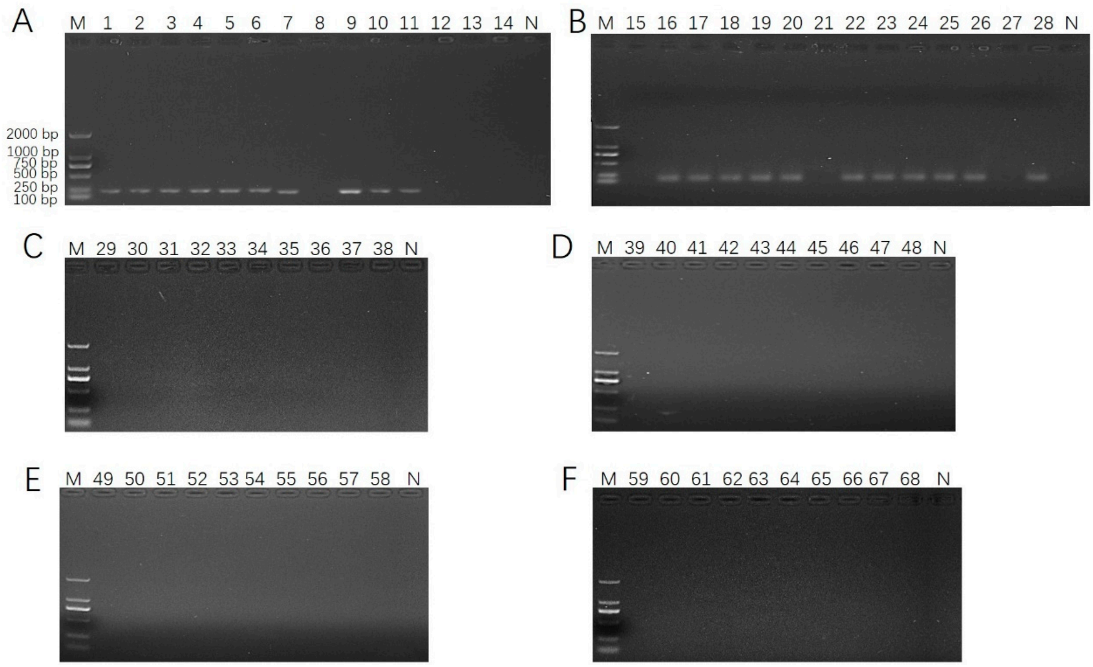

Figure S1. *E. epinepheli* detection results conducted by PCR in 68 samples collected from grouper farms in Hainan, China. Numbers 1 to 14 correspond to the tissue samples labeled T1 to T14 in Table S2 (A); Numbers 15 to 28 correspond to the tissue samples labeled T15 to T28 in Table S2 (B); Numbers 29 to 38 correspond to the tissue samples labeled T29 to T38 in Table S2 (C); Numbers 39 to 42 correspond to the tissue samples labeled T39 to T42 in Table S2, while numbers 43 to 48 correspond to the water samples labeled W1 to W6 in Table S3 (D); Numbers 49 to 58 correspond to the water samples labeled W7 to W16 in Table S3 (E); Numbers 59 to 68 correspond to the water samples labeled W17 to W26 in Table S3 (F). N, Negative control.

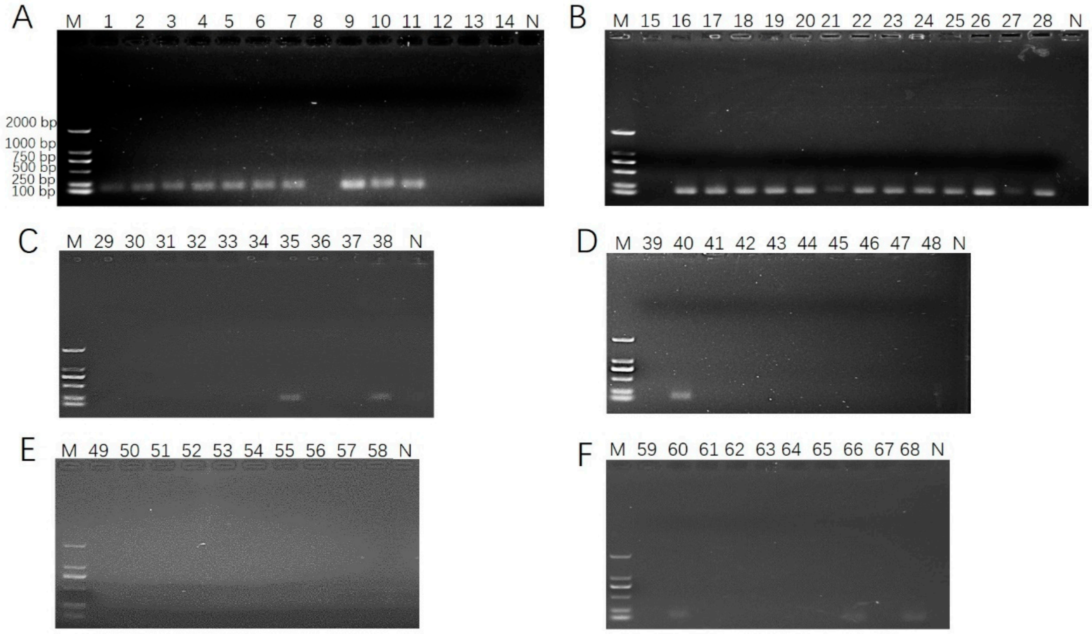

Figure S2. *E. epinepheli* detection results conducted by ERA in 68 samples collected from grouper farms in Hainan, China. Numbers 1 to 14 correspond to the tissue samples labeled T1 to T14 in Table S2 (A); Numbers 15 to 28 correspond to the tissue samples labeled T15 to T28 in Table S2 (B); Numbers 29 to 38 correspond to the tissue samples labeled T29 to T38 in Table S2 (C); Numbers 39 to 42 correspond to the tissue samples labeled T39 to T42 in Table S2, while numbers

43 to 48 correspond to the water samples labeled W1 to W6 in Table S3 (D); Numbers 49 to 58 correspond to the water samples labeled W7 to W16 in Table S3 (E); Numbers 59 to 68 correspond to the water samples labeled W17 to W26 in Table S3 (F). N, Negative control.

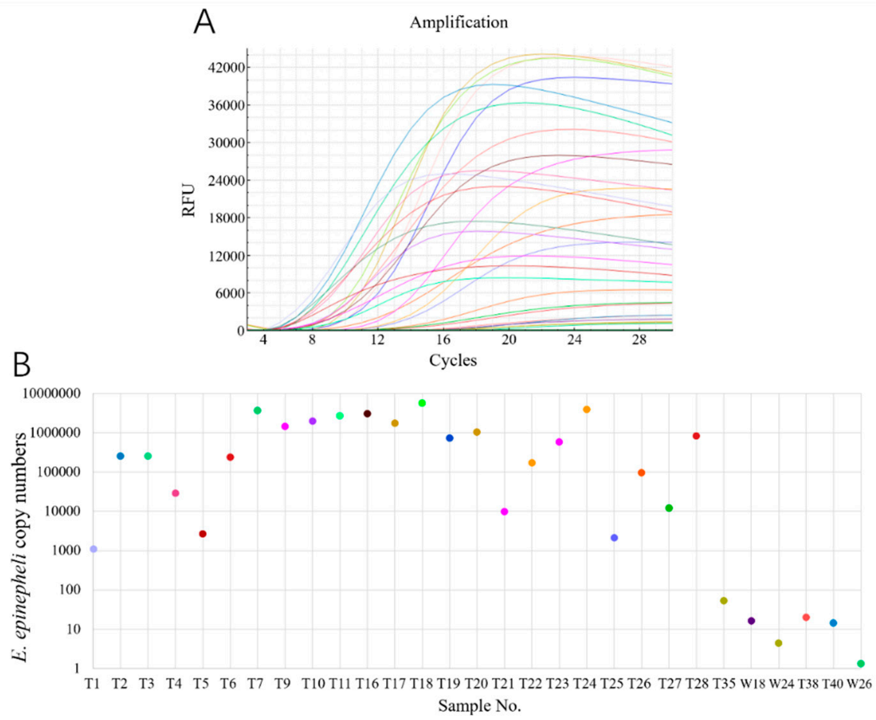

Figure S3. *E. epinepheli* detection results conducted by RT-ERA. Detection results of *E. epinepheli* in 68 samples collected from grouper farms in Hainan, China. (A); Copy numbers of *E. epinepheli* in 29 positive samples calculated by standard curve (B). Sample numbers in Figure 8B correspond to samples labeled in Table S2 and Table S3. N, Negative control.

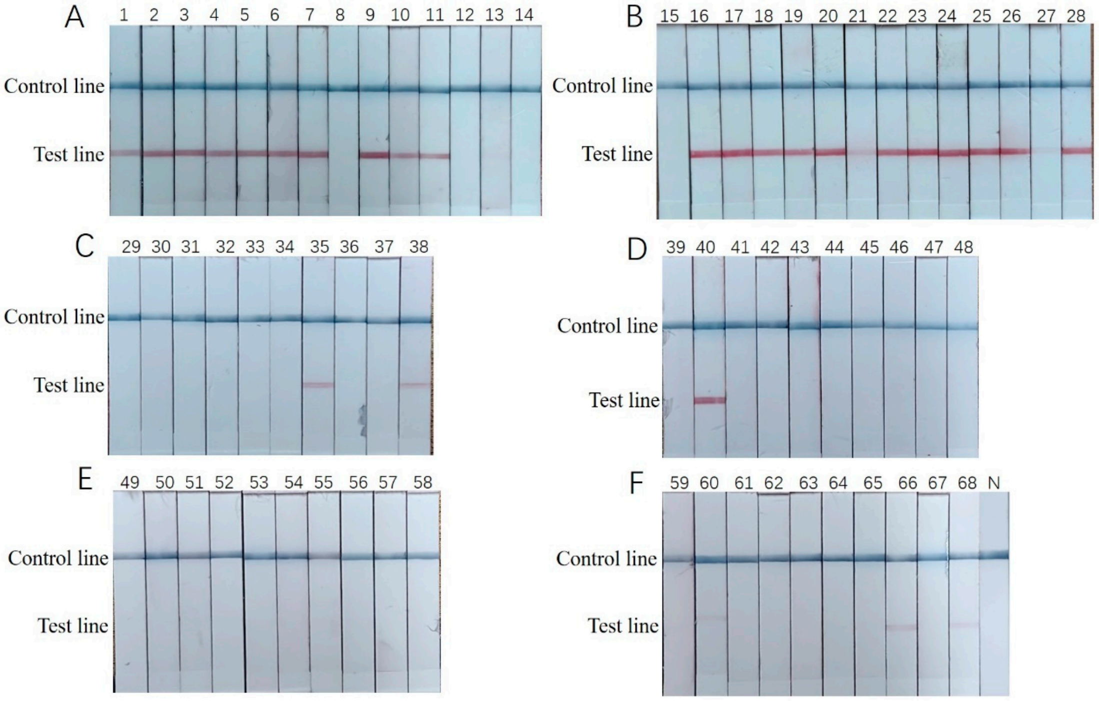

Figure S4. *E. epinepheli* detection results conducted by ERA-LFD in 68 samples collected from grouper farms in Hainan, China. Numbers 1 to 14 correspond to the tissue samples labeled T1 to T14 in Table S2 (A); Numbers 15 to 28 correspond

to the tissue samples labeled T15 to T28 in Table S2 (B); Numbers 29 to 38 correspond to the tissue samples labeled T29 to T38 in Table S2 (C); Numbers 39 to 42 correspond to the tissue samples labeled T39 to T42 in Table S2, while numbers 43 to 48 correspond to the water samples labeled W1 to W6 in Table S3 (D); Numbers 49 to 58 correspond to the water samples labeled W7 to W16 in Table S3 (E); Numbers 59 to 68 correspond to the water samples labeled W17 to W26 in Table S3 (F). N, Negative control.

Supplementary File S1 The *E. epinepheli* 18S rRNA sequence related information

A: Four 18S rRNA Sequences of E. epinepheli retrieved from GenBank

>MH345732

AACTAACCACGGTAACCTGTGGCTAAAAATTTAGAATAAGGCGCAGTCCTATTATGTTTGTTGGTAGTGT
AATGGACTCCCAAGGCAACAATGGGTGACGGGAAATCAGGGTTTGATTCCGGAGAGGGAGCCTGAGAGAT
GGCTCCCACGTCCAAGGACGGCAGCAGGCGCGAAAATTGTCCACTTTTTTGAGAAGAGACATTTATTAGA
CGTGACTAAAAGAGCGTAGTTTAAAAAACTATGTGTAAAGTTATTGGAGGGTAAGTTTGGTGCCAGCAGC
CGCGGTAATTCCGACTCCAAGAGTGTTCATGATGGTTGTTGCAGTTAAAACGTCCGTAGCTGTAGATGCA
ATTAAAACACGATGATAAAAATCGTAGAGTTTGCTAAATATTGCTAGCGGAATGGATAGGGAACGTGGTA
TAGGTAGACAAAGAATGAAATCTCTAGACTCTACCTGGACCAACAGAGGCGAAAGCGACGTTCTTAGACG
TATCCGAGGGTCAAGGACTAAGGCTGGAGTATTGAAAGTGATTAGATACCGCTGTAATTCTAGCAGTAAA
CTATGCCGACAATTTTTAGTGACTTTTTAAAAAGTTTGCTGGGAATAAGAGAAATCAAGTTTTCGGGCTC
TGGGGATAGTACAGCCGCAAGACTGAAACTTAAACGATATTGGCGGAAGTTTACCACCAGTAGTGGATTA
TGCCGCTTAATTTAACTCAACGCGGGAAAACTTACCAGGGTCAAGTTTATCGTAGATTGATTACATGAGA
TAAACGAGAGTGGTGCATGGCCGTTGTAAATTGATGGAATGATCTTTAACTTAAGTGTTGGAATCAGTGA
GATCTATGAGACAGGTGCTTTTTAAGTACAGGAAGGTGTAGACGATAACAGGTCCGTAATGCCCTCAGAT
ATCCTGGGCAGCAAGCGTAATACAATATCACTTAAAAAAATAAAGAAATTTGTGATAAGCAGGATTAGCT
TTTGTAAATAAGCTATGAACGAGGAATTACTAGTAACGGTGCCTCATCAAGGCATGGTGAATG

>KR263870

AACTAACCACGGTAACCTGTGGCTAAAAATTTAGAATAAGGCGCAGTCCTATTATGTTTGTTGGTAGTGT
AATGGACTCCCAAGGCAACAATGGGTGACGGGAAATCAGGGTTTGATTCCGGAGAGGGAGCCTGAGAGAT
GGCTCCCACGTCCAAGGACGGCAGCAGGCGCGAAAATTGTCCACTTTTTTGAGAAGAGACATTTATTAGA
CGTGACTAAAAGAGCGTAGTTTAAAAAACTATGTGTAAAGTTATTGGAGGGTAAGTTTGGTGCCAGCAGC
CGCGGTAATTCCGACTCCAAGAGTGTTCATGATGGTTGTTGCAGTTAAAACGTCCGTAGCTGTAGATGCA
ATTAAAACACGATGATAAAAATCGTAGAGTTTGCTAAATATTGCTAGCGGAATGGATAGGGAACGTGGTA
TAGGTAGACAAAGAATGAAATCTCTAGACTCTACCTGGACCAACAGAGGCGAAAGCGACGTTCTTAGACG
TATCCGAGGGTCAAGGACTAAGGCTGGAGTATTGAAAGTGATTAGATACCGCTGTAATTCTAGCAGTAAA
CTATGCCGACAATTTTTAGTGACTTTTTAAAAAGTTTGCTGGGAATAAGAGAAATCAAGTTTTCGGGCTC
TGGGGATAGTACAGCCGCAAGACTGAAACTTAAACGATATTGGCGGAAGTTTACCACCAGTAGTGGATTA
TGCCGCTTAATTTAACTCAACGCGGGAAAACTTACCAGGGTCAAGTTTATCGTAGATTGATTACATGAGA
TAAACGAGAGTGGTGCATGGCCGTTGTAAATTGATGGAATGATCTTTAACTTAAGTGTTGGAATCAGTGA
GATCTATGAGACAGGTGCTTTTTAAGTACAGGAAAGGTGTAGGACGATAACAGGTCCGTAATGCCCTCAG
ATATCCTGGGCAGCAAGCGTAATACAATATCACTTAAAAAAATAAAGAAATTTGTGATAAGCAGGATTAG
CTTTTGTAAATAAGCTATGAACGAGGAATTACTAGTAACGGTGCCTCATCAAGGCATGGTGAATG

>OR143128

CACCAGGTTGATTCTGCCCGAAATGAATGCTAGTCTTTAAGATTAAGCCATGTATGTTAAGCGAACCTGA
ACAGGGGAGCGACAAACGGCTCAGTAATGCTGCGCTTATTTGCTCTAAATGTAAAAACTAACCACGGTAA
CCTGTGGCTAAAAATTTAGAATAAGGCGCAGTCCTATTATGTTTGTTGGTAGTGTAATGGACTCCCAAGG
CAACAATGGGTGACGGGAAATCAGGGTTTGATTCCGGAGAGGGAGCCTGAGAGATGGCTCCCACGTCCAA

GGACGGCAGCAGGCGCGAAAATTGTCCACTTTTTTGAGAAGAGACATTTATTAGACGTGACTAAAAGAGC
GTAGTTTAAAAAACTATGTGTAAAGTTATTGGAGGGTAAGTTTGGTGCCAGCAGCCGCGGTAATTCCGAC
TCCAAGAGTGTTCATGATGGTTGTTGCAGTTAAAACGTCCGTAGCTGTAGATGCAATTAAAACACGATGA
TAAAAATCGTAGAGTTTGCTAAATATTGCTAGCGGAATGGATAGGGAACGTGGTATAGGTAGACAAAGAA
TGAAATCTCTAGACTCTACCTGGACCAACAGAGGCGAAAGCGACGTTCTTAGACGTATCCGAGGGTCAAG
GACTAAGGCTGGAGTATTGAAAGTGATTAGATACCGCTGTAATTCTAGCAGTAAACTATGCCGACAATTT
TTAGTGACTTTTTAAAAAGTTTGCTGGGAATAAGAGAAATCAAGTTTTCGGGCTCTGGGGATAGTACAGC
CGCAAGACTGAAACTTAAACGATATTGGCGGAAGTTTACCACCAGTAGTGGATTATGCCGCTTAATTTAA
CTCAACGCGGGAAAACTTACCAGGGTCAAGTTTATCGTAGATTGATTACATGAGATAAACGAGAGTGGTG
CATGGCCGTTGTAAATTGATGGAATGATCTTTAACTTAAGTGTTGGAATCAGTGAGATCTATGAGACAGG
TGCTTTTTAAGTACAGGAAGGTGTAGACGATAACAGGTCCGTAATGCCCTCAGATATCCTGGGCAGCAAG
CGTAATACAATATCACTTAAAAAAATAAAGAAATTTGTGATAAGCAGGATTAGCTTTTGTAAATAAGCTA
TGAACGAGGAATTACTAGTAACGACGTCTCATCAAGGCGTGGTGAATGTGTCCCTGAACTTTGCACACAC
CGCCCGTCACTAACTCAGATGGCTATAGGGGTGAAGAGCTACGGTTCTGAACATCTATAGCTAGATGATT
TAAAAGTCGCAACAAGGTAACTGTAGGTGAACCTGCAGTTGGATCATTAACAAAAAAAGATTTTTTTGTC
TAGATACTGTTTGTATATTTATGCAATTTATCAGAATAAATAGATTGTTACATTGCACACACATTAAGTA
GTATATTTATGTTTATTATCAAATTTTAAGCATGTTTGTCATCTTCTTTTATCTTTTTACAATTTTCAAT
ATTTTTATCATTCTATCTTATATACACTATACTAATAAGAAAGAAACAAAAAAATTTCATAAAAATAAAA
ACATCTATCTTTTACAATAATGTTTATCTACAATTACACAACACTTCTTTTACTACTACTTTATTATTCT
AAATTGTAAACATCTATTTTCAATTATCTTTATTTATATATTACCCACTATATACATTTTTTGACCATAT
ATTTATAGGAGCAGTAATAAGCACTAAATATAAAAAAATAAAAAAATTTAGATAAGTATCTCTAGTACAG
GATCATTTGGGTCTAACAGCGATGACGAACCCAAAAGACTGGGAAAAGTTTAACGACTATTCTAATCATG
AAGGTTCAACTTCAAGGTGACCTCCTGAACTTAAGCATATCACTAAGGAGAGGAAAATAAACTAACTAGG
ATTTCATTAGTAGTGGCGAATGAACATGAAAAAGCTCTATTGGTAAAAAACAAATTTATTTTGTTTTATT
GTAAAATAGAAAAAGGGATAACAATAATTCTGTTGGAAAACAGTGCCACCGTGGGTGATAGCCCCAAATA
AAATCCTACAAAAATATTTAAAGAGTAGTGCTGTTTGGTAGTGCAGTATGAAAAGGAGGTAGCAACTTCT
AAAGCTAAATATAGTTAGATACCGATAGCAAAGAAGTAGCACGAGCGAAAATTGAAAAATTCTCATCTAG
AGAGTGAAATAGTGAAAGTACTAGAGTGTAAGCCGAGTATTTCGGACCCGTATTGAAACACGGACCAAGG
AGAA
>OR238515
CACCAGGTTGATTCTGCCCGAAATGAATGCTAGTCTTTAAGATTAAGCCATGCATGTTAAGCGAACCTGA
ACAGGGGAGCGACAAACGGCTCAGTAATGCTGCGCTTATTTGCTCTAAATGTAAAAACTAACCACGGTAA
CCTGTGGCTAAAAATTTAGAATAAGGCGCAGTCCTATTATGTTTGTTGGTAGTGTAATGGACTCCCAAGG
CAACAATGGGTGACGGGAAATCAGGGTTTGATTCCGGAGAGGGAGCCTGAGAGATGGCTCCCACGTCCAA
GGACGGCAGCAGGCGCGAAAATTGTCCACTTTTTTGAGAAGAGACATTTATTAGACGTGACTAAAAGAGC
GTAGTTTAAAAAACTATGTGTAAAGTTATTGGAGGGTAAGTTTGGTGCCAGCAGCCGCGGTAATTCCGAC
TCCAAGAGTGTTCATGATGGTTGTTGCAGTTAAAACGTCCGTAGCTGTAGATGCAATTAAAACACGATGA
TAAAAATCGTAGAGTTTGCTAAATATTGCTAGCGGAATGGATAGGGAACGTGGTATAGGTAGACAAAGAA
TGAAATCTCTAGACTCTACCTGGACCAACAGAGGCGAAAGCGACGTTCTTAGACGTATCCGAGGGTCAAG
GACTAAGGCTGGAGTATTGAAAGTGATTAGATACCGCTGTAATTCTAGCAGTAAACTATGCCGACAATTT
TTAGTGACTTTTTAAAAAGTTTGCTGGGAATAAGAGAAATCAAGTTTTCGGGCTCTGGGGATAGTACAGC
CGCAAGACTGAAACTTAAACGATATTGGCGGAAGTTTACCACCAGTAGTGGATTATGCCGCTTAATTTAA
CTCAACGCGGGAAAACTTACCAGGGTCAAGTTTATCGTAGATTGATTACATGAGATAAACGAGAGTGGTG
CATGGCCGTTGTAAATTGATGGAATGATCTTTAACTTAAGTGTTGGAATCAGTGAGATCTATGAGACAGG

TGCTTTTTAAGTACAGGAAGGTGTAGACGATAACAGGTCCGTAATGCCCTCAGATATCCTGGGCAGCAAG
CGTAATACAATATCACTTAAAAAAATAAAGAAATTTGTGATAAGCAGGATTAGCTTTTGTAAATAAGCTA
TGAACGAGGAATTACTAGTAACGACGTCTCATCAAGGCGTGGTGAATGTGTCCCTGAACTTTGCACACAC
CGCCCGTCACTAACTCAGATGGCTATAGGGGTGAAGAGCTACGGTTCTGAACATCTATAGCTAGATGATT
TAAAAGTCGCAACAAGGTAACTGTAGGTGAACCTGCAGTTGGATCATTAACAAAAATGATTTTTTTGTCT
AGATACTGTTTGTATATTTATGCAATTTATCAGAATAAATAGATTTTTACATTTTAACAAACATCAAGTA
GTATATAAATGTTTATTATCAAATTTTAAGCATTTTTCTCATCTTCTTTTTTATTTTTACAATTTTCAAT
ATTTTTATCATTCTATTCTTATATACACTATACTAATAAGAAAGAAACAAAAAAAATTTCATAAAAATAAA
AACATCTATCTTTTACAATAATGTTTATCTACAATTACACAACACTTCTTTTACTACTACTTTATTATTT
TAAATTGTAAACATCTATTTTCAATTATCTTTATTTTTATTACTACCCACTATATACATTTTTTAACCAT
ATATTTATAGGAGCAGTAATAAGCACTAAATATAAAAAATAAAAAAAATTTAGATAAGTATCTCTAGTACA
GGATCATTTGGGTCTAACAGCGATGACGAACCCAAAAGACTGGGAAAAGTTTAACGACTATTCTAATCAT
GAAGGTTCAACTTCAAGGTGACCTCCTGAACTTAAGCATATCACTAAGGAGAGGAAAATAAACTAACTAG
GATTTCATTAGTAGTGGCGAATGAACATGAAAAAGCTCTATTGGTAAAAAACAAATTTATTTTGTTTTAT
TGTAAAATAGAAAAAGGGATAACAATAATTCTGTTGGAAAACAGTGCCACCGTGGGTGATAGCCCCAAAT
AAAATCCTACAAAAATATTTAAAGAGTAGTGCTGTTTGGTAGTGCAGTATGAAAAGGAGGTAGCAACTTC
TAAAGCTAAATATAGTTAGATACCGATAGCAAAGAAGTAGCACGAGCGAAAATTGAAAAATTCTCATCTA
GAGAGTGAAATAGTGAAAGTACTAGAGTGTAAGCCGAGTATTTCGGACCCGTATTGAAACACGGACCAAG
GAGA

B: Multiple sequence alignment results of four 18S rRNA gene of E. epinepheli **performed by NCBI blastn**

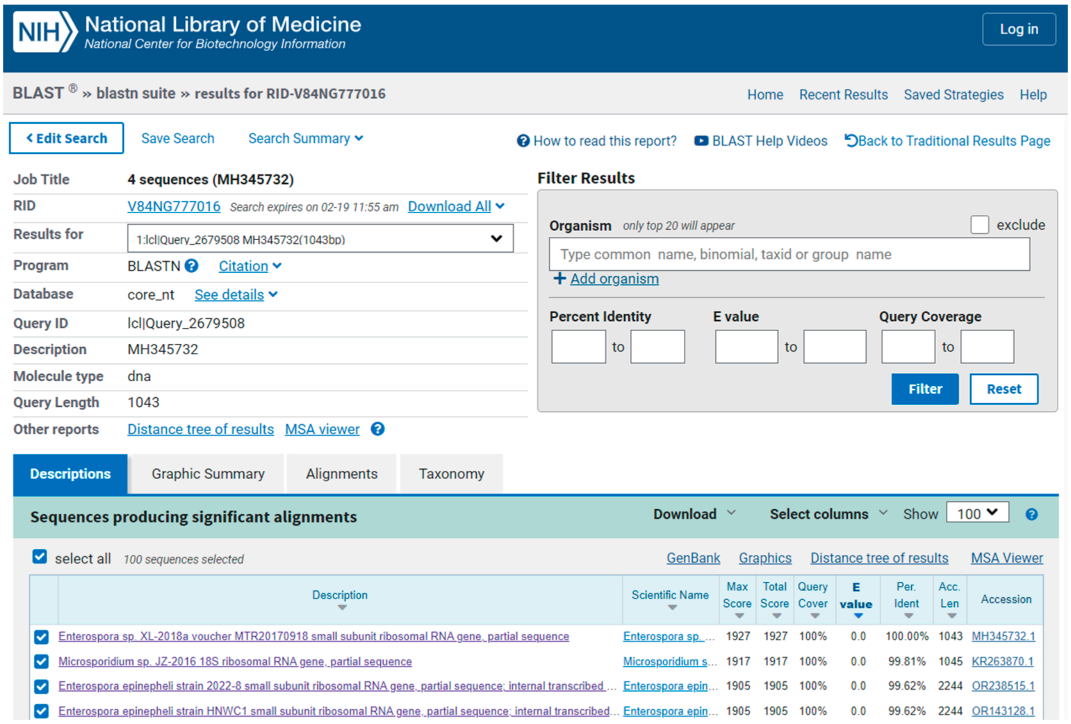

C: The identity of the selected primer sets with four 18S rRNA gene of E. epinepheli performed by Clustal Omega. Note: FP13 and RP 13 are the final selected forward and reverse primer set (No. 13 in table S1) of this study. 'RP13 converted' refers to the reverse primer (RP13) being reverse-complemented for alignment.

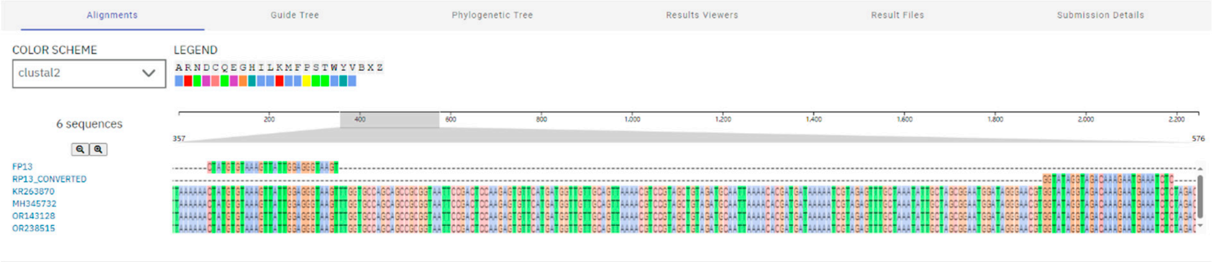

If you use this service, please consider citing the following publication: The EMBL-EBI Job Dispatcher sequence analysis tools framework in 2024. More information about this bioinformatics application can be found in its bio.tools record.

```
FP13              ------------------------------------------------------------    0
RP13_converted    ------------------------------------------------------------    0
KR263870          ATTGTCCACTTTTTTGAGAAGAGACATTTATTAGACGTGACTAAAAGAGCGTAGTTTAAA    235
MH345732          ATTGTCCACTTTTTTGAGAAGAGACATTTATTAGACGTGACTAAAAGAGCGTAGTTTAAA    235
OR143128          ATTGTCCACTTTTTTGAGAAGAGACATTTATTAGACGTGACTAAAAGAGCGTAGTTTAAA    360
OR238515          ATTGTCCACTTTTTTGAGAAGAGACATTTATTAGACGTGACTAAAAGAGCGTAGTTTAAA    360

FP13              ---CTATGTGTAAAGTTATTGGAGGGTAAGT-----------------------------    28
RP13_converted    ------------------------------------------------------------    0
KR263870          AAACTATGTGTAAAGTTATTGGAGGGTAAGTTTGGTGCCAGCAGCCGCGGTAATTCCGAC    295
MH345732          AAACTATGTGTAAAGTTATTGGAGGGTAAGTTTGGTGCCAGCAGCCGCGGTAATTCCGAC    295
OR143128          AAACTATGTGTAAAGTTATTGGAGGGTAAGTTTGGTGCCAGCAGCCGCGGTAATTCCGAC    420
OR238515          AAACTATGTGTAAAGTTATTGGAGGGTAAGTTTGGTGCCAGCAGCCGCGGTAATTCCGAC    420

FP13              ------------------------------------------------------------    28
RP13_converted    ------------------------------------------------------------    0
KR263870          TCCAAGAGTGTTCATGATGGTTGTTGCAGTTAAAACGTCCGTAGCTGTAGATGCAATTAA    355
MH345732          TCCAAGAGTGTTCATGATGGTTGTTGCAGTTAAAACGTCCGTAGCTGTAGATGCAATTAA    355
OR143128          TCCAAGAGTGTTCATGATGGTTGTTGCAGTTAAAACGTCCGTAGCTGTAGATGCAATTAA    480
OR238515          TCCAAGAGTGTTCATGATGGTTGTTGCAGTTAAAACGTCCGTAGCTGTAGATGCAATTAA    480

FP13              ------------------------------------------------------------    28
RP13_converted    ------------------------------------------------------------    0
KR263870          AACACGATGATAAAAATCGTAGAGTTTGCTAAATATTGCTAGCGGAATGGATAGGGAACG    415
MH345732          AACACGATGATAAAAATCGTAGAGTTTGCTAAATATTGCTAGCGGAATGGATAGGGAACG    415
OR143128          AACACGATGATAAAAATCGTAGAGTTTGCTAAATATTGCTAGCGGAATGGATAGGGAACG    540
OR238515          AACACGATGATAAAAATCGTAGAGTTTGCTAAATATTGCTAGCGGAATGGATAGGGAACG    540

FP13              ------------------------------------------------------------    28
RP13_converted    -GGTATAGGTAGACAAAGAATGAAATCTC-------------------------------    28
KR263870          TGGTATAGGTAGACAAAGAATGAAATCTCTAGACTCTACCTGGACCAACAGAGGCGAAAG    475
MH345732          TGGTATAGGTAGACAAAGAATGAAATCTCTAGACTCTACCTGGACCAACAGAGGCGAAAG    475
OR143128          TGGTATAGGTAGACAAAGAATGAAATCTCTAGACTCTACCTGGACCAACAGAGGCGAAAG    600
OR238515          TGGTATAGGTAGACAAAGAATGAAATCTCTAGACTCTACCTGGACCAACAGAGGCGAAAG    600

FP13              ------------------------------------------------------------    28
RP13_converted    ------------------------------------------------------------    28
KR263870          CGACGTTCTTAGACGTATCCGAGGGTCAAGGACTAAGGCTGGAGTATTGAAAGTGATTAG    535
MH345732          CGACGTTCTTAGACGTATCCGAGGGTCAAGGACTAAGGCTGGAGTATTGAAAGTGATTAG    535
OR143128          CGACGTTCTTAGACGTATCCGAGGGTCAAGGACTAAGGCTGGAGTATTGAAAGTGATTAG    660
OR238515          CGACGTTCTTAGACGTATCCGAGGGTCAAGGACTAAGGCTGGAGTATTGAAAGTGATTAG    660
```
